# Curcumin in Osteosarcoma Therapy: Combining With Immunotherapy, Chemotherapeutics, Bone Tissue Engineering Materials and Potential Synergism With Photodynamic Therapy

**DOI:** 10.3389/fonc.2021.672490

**Published:** 2021-05-20

**Authors:** Chunfeng Xu, Mingjie Wang, Wei Guo, Wei Sun, Yuelian Liu

**Affiliations:** ^1^ Section of Restorative and Reconstructive Oral Care, Department of Oral Health Sciences, Academic Center for Dentistry Amsterdam (ACTA), University of Amsterdam and Vrije Universiteit Amsterdam, Amsterdam, Netherlands; ^2^ Department of Oral–Maxillofacial and Head–Neck Oncology, Shanghai Ninth People’s Hospital, Shanghai Jiaotong University School of Medicine, Shanghai, China; ^3^ Department of Mechanical Engineering, Drexel University, Philadelphia, PA, United States; ^4^ Department of Mechanical Engineering, Tsinghua University, Beijing, China

**Keywords:** osteosarcoma, curcumin, immunotherapy, chemotherapy, bone tissue engineering, biomaterials, photodynamic therapy

## Abstract

Osteosarcoma is a dominating malignant bone tumor with high mortality due to pulmonary metastases. Furthermore, because of the cancer cell erosion and surgery resection, osteosarcoma always causes bone defects, which means dysfunction and disfigurement are seldom inevitable. Although various advanced treatments (e.g. chemotherapy, immunotherapy, radiotherapy) are coming up, the 5-year survival rate for osteosarcoma with metastases is still dismal. In line with this, the more potent treatments for osteosarcoma are in high demand. Curcumin, a perennial herb, has been reportedly applied in the therapy of various types of tumors via different mechanisms. *In vitro*, it has also been reported that curcumin can inhibit the proliferation of osteosarcoma cell lines and can be used to repair bone defects. This seems curcumin is a promising candidate in osteosarcoma treatment. However, due to its congenital property like hydrophobicity, and low bioavailability, affecting its anticancer effect, clinical applications of curcumin are highly limited. To enhance its performance in cancer therapies, some synergist approaches with curcumin have emerged. The present review presents some prospective ones (i.e. combinations with immunotherapy, chemotherapeutics, bone tissue engineering, and biomaterials) applied in osteosarcoma treatment. Additionally, with the advancements of photodynamic therapy in cancer therapy, this review also prospects the combination of curcumin with photodynamic therapy in osteosarcoma treatment.

## Introduction

Osteosarcoma (OS) originating from mesenchymal stem cells is the main primary malignant bone tumor (1), making up for *ca*. 35% of all bone carcinomas (2); it is usually diagnosed in children and adolescents (3). The principal cause of death in patients suffering from OS is pulmonary metastases (4). More than 90% of these patients died from this before the introduction of polychemotherapy (5). Another reason for the high mortality may refer to the rapid tumor development: frustratingly, once diagnosed, OS has most been in stage IIb or III (6). Furthermore, bone metastases are also common in OS, causing bone defects and followed by potential dysfunction and disfigurement (7, 8). However, to date, it is still hard to identify a targeted treatment for OS, as it is with a high frequency of gene and chromosome mutations (9). Currently, the prevailing remedies for OS are surgery, neoadjuvant and adjuvant chemotherapy. Conventionally, OS is indicated to be resistant to radiotherapy, nonetheless, it is implied that it is beneficial for those who have received chemotherapy but are unable to undergo complete resection (10). With these modern systemic therapies, the 5-year survival rate has improved, while this rate of those with metastases is still dismal—less than 30% (8). On the other hand, in the latest decades, therapeutic approaches for OS have not developed. Regarding this, more efficient therapies are still in urgent need.

**Figure 1:**
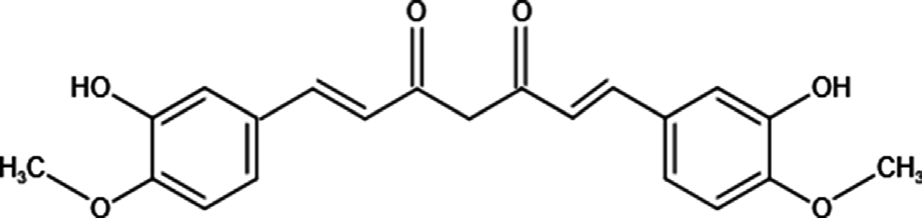
Chemical structure of curcumin.

Curcumin also named 1,7-Bis(4-hydroxy-3-methoxyphenyl)-1,6-heptadiene-3,5-dione, a natural polyphenol, is isolated from the rhizome of *Curcuma longa* (11). Although curcumin is isolated from herbs, its chemical structure has been identified (**Figure 1**). Structurally, there are 3 reactive sites in curcumin: metal chelator, Michael acceptor, and hydrogen atom donor, which bestows versatile abilities on curcumin to fight against diseases. It has been reported that curcumin possesses not only anti-inflammatory, anti-oxidative but also anti-tumor potential through targeting various molecules (12–16). In cancer treatments, curcumin suppresses tumor progression *via* various mechanisms (**Table 1**); commercial curcumin products have been used to evaluate the anti-cancer effect *in vitro* and *in vivo* (31). As a capable phytochemical, it has identified curcumin inhibits the proliferation of osteosarcoma cell lines and induces their apoptosis (24, 32, 33). Moreover, curcumin is with the potential to repair bone defects owing to tumor erosion or surgery (34–37). Taken together, curcumin seems to be an outstanding candidate that can be used in osteosarcoma treatment with the “one stone two birds” effect: inhibiting OS progression and repairing the bone defects simultaneously. Nevertheless, due to its poor aqueous solubility—about 11 ng/ml in water (38), rapid metabolism, and rapid system elimination, contributing to the low bioavailability (39), its clinical applications are not common currently. Based on some research, it has been demonstrated that the IC50 of curcumin for most cancer cells is 15–30 μM, whereas, the highest concentration of curcumin in the human body is just in the nanomolar range (40). Hence, to improve its anti-cancer efficacy, synergistic approaches have been carried out. We herein summarize combinations of free curcumin with other therapeutic strategies to enhance its anticancer effect on OS treatments.

**Table 1:** Targets of curcumin in anticancer treatments.

Targets of Curcumin	
Breast Cancer	NF-κB (17), Nrf2 (18), MMPs, VEGF (19), Akt (20)
Lung Cancer	PI3K/Akt/mTOR (21), EGFR and TLR4/MyD88 (22)
Osteosarcoma	p-JAK2/p-STAT3 (23), Notch-1 (24), miR-138 (25)
Head & Neck Cancer	IL-6/p-STAT3 (26), NF-κB, cyclin D1, and Bcl-2 (27)
Gastric Cancer	PI3K and P53 (28), ROS (29), Wnt/β-catenin (30)

## Inhibition Effect of Curcumin on Tumors

**Figure 2:**
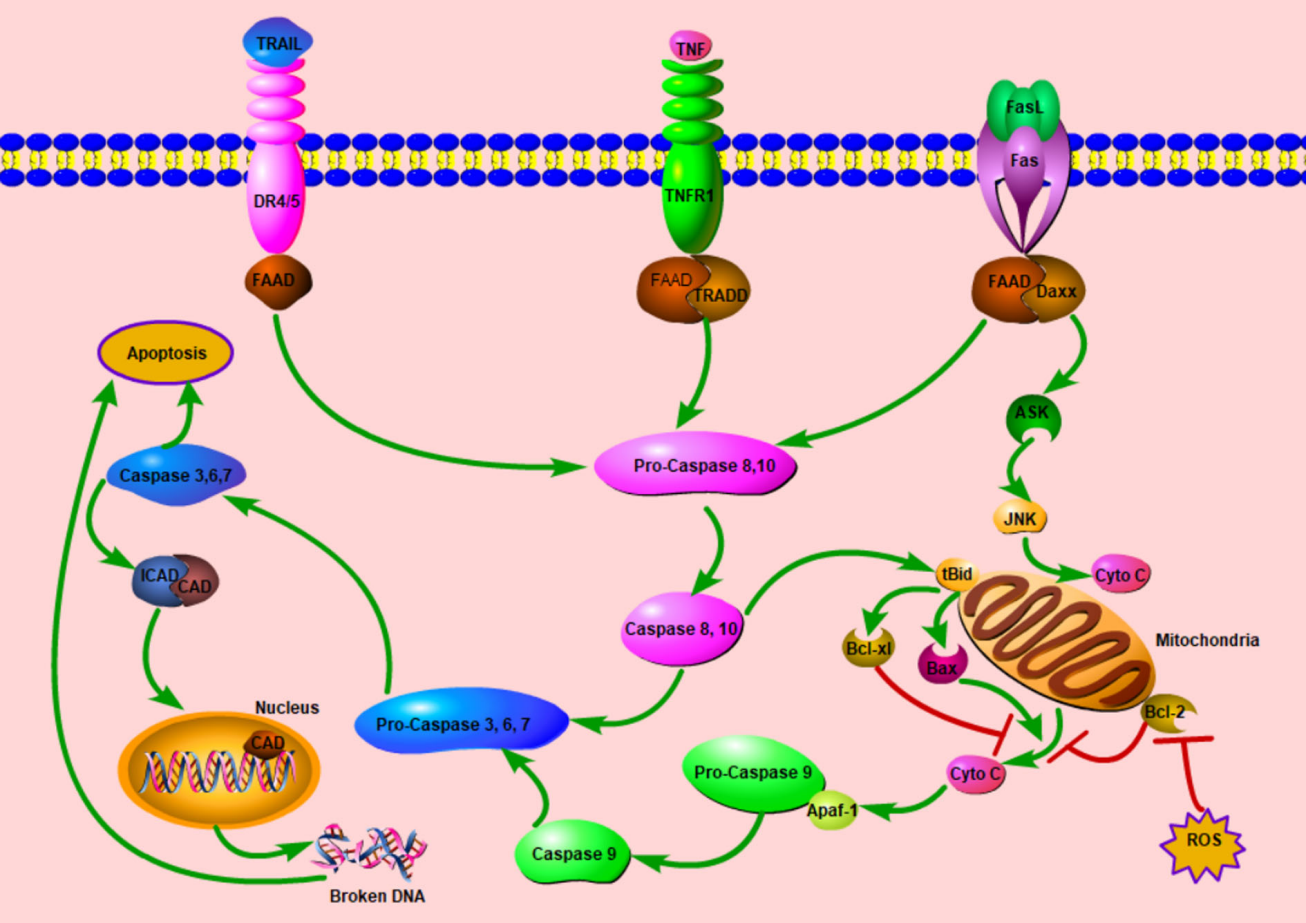
Schematic diagram of cell apoptosis. Once the extracellular cytokines bond to relative death receptors (DRs), DRs recruit FADD intracellularly, this complex initiates the cascade of apoptosis. In some stress conditions, mitochondrial permeability transition will increase, afterwards, Cyto C will be released. With Apaf-1, Cyto C will start the activation cascade from Pro-Caspase 9. In these two ways, caspase 3 is activated finally and exerts the apoptosis process with various mechanisms including the DNA fragmentation in the nucleus. The extrinsic pathway can modulate the intrinsic pathway by the tBid, ASK, and JNK. ASK, apoptosis signal-regulating kinase; Apaf-1, apoptotic protease activating factor-1; CAD, caspase-activated deoxyribonuclease; Cyto C; cytochrome C; Daxx, death domain associated protein; DR, death receptor; FAAD, Fas-associated death domain protein; ICAD, inhibitor of caspase-activated deoxyribonuclease; JNK, c-Jun N-terminal kinase; tBid, truncated Bid; ROS, reactive oxygen species; TNF, tumor necrosis factor; TNFR1, tumor necrosis factor receptor 1; TRADD, Tumor necrosis factor receptor type 1-associated death domain protein. TRAIL, tumor necrosis factor related apoptosis inducing ligand; **┤**, Inhibition; →, Promotion.

Apoptosis or programmed cell death (PCD) plays a potent role in tumorigenesis. In physiological conditions, it can eliminate the precancerous cells, thereby preventing normal cells from being malignant; in turn, anticancer agents will induce cancer cells apoptosis to cure cancers. Generally, there are two canonical apoptotic pathways: extrinsic and intrinsic pathways (**Figure 2**). For the former, apoptosis initiates after the bond between some extracellular cytokines or growth factors and their receptors, death receptors, on the cytomembrane, which will activate caspase 8 followed by the activation of caspase 3 finally. The well-known death receptor couples are TNF-TNFR1 and FasL-Fas (41). For the intrinsic pathway, apoptosis is mainly induced by the mitochondria dysfunction attributed to some stress conditions. With increased mitochondria membrane potential, some molecules (mainly cytochrome c) released from mitochondria will initiate the process of apoptosis.

Curcumin can inhibit cancer development via various mechanisms: inducing apoptosis and some miRNA expression, dampening angiogenesis, metastasis, etc. Curcumin is identified to induce neoplasm apoptosis through extrinsic and intrinsic pathways *via* various targets such as Bax, Bcl-2, Fas, p53 (42–44). It is also found to suppress non-small cell lung cancer by upregulating miR192-5p (45) and in leukemic cells, curcumin can upregulate miR-15a and miR-16-1, which will decrease WT-1expression, thereby suppressing the proliferation of leukemic cells (46).

For osteosarcoma, several researchers have successfully proven that curcumin can induce the MG63, U2OS, and HOS cell line apoptosis based on different signal pathways (32, 33, 47–50). Besides, curcumin has also been identified to suppress the proliferation, invasion, and metastasis of osteosarcoma (23, 24, 51, 52). Thence, curcumin is a promising agent with multifaced roles it plays in the treatment of osteosarcoma. However, due to poor bioavailability, the administration of curcumin in cancer treatment is not common. To overcome this issue and improve its efficiency in tumor therapy, synergistic approaches are carried out.

## Synergistic Approaches

### Combination With Immunotherapy

**Figure 3:**
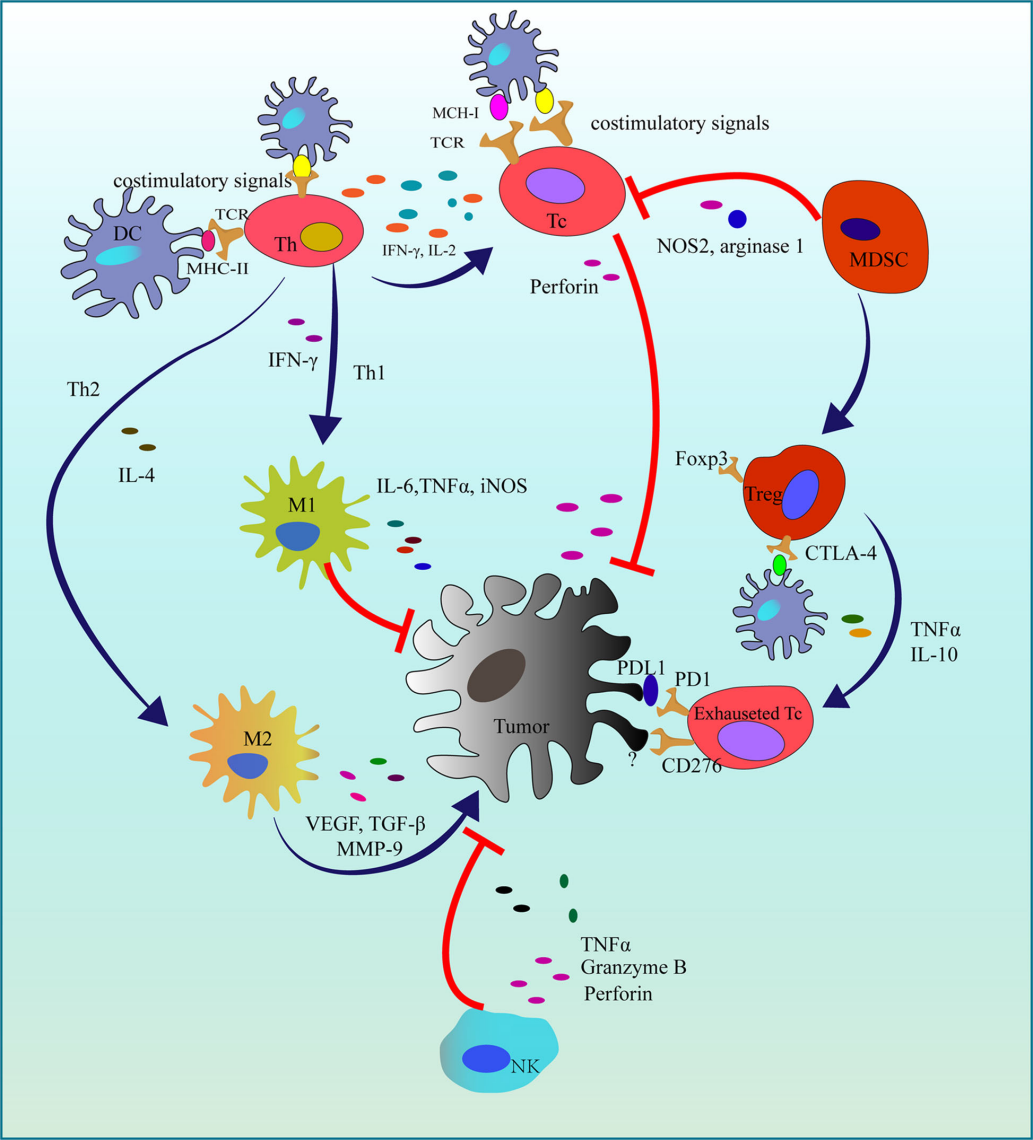
Immune responses in the tumor microenvironment. In the initiation of tumorigenesis, activated Tc and NK cells suppress tumor proliferation. While in the immune evasion, Th2 recruits MDSC and promotes macrophages polarization (M1 to M2). Finally, survived tumor cells exhaust Tc cells by some immune checkpoints. DC, Dendritic cell; MCH-1, Major histocompatibility complex class I; MCH-II, Major histocompatibility complex class II; TCR, T cell receptor; Th, T helper cell; Th1, T helper 1 cell; Th2, T helper 2 cell; Tc, Cytotoxic T cell; IFN-γ, Interferon-gamma; Treg, Regulatory T cell; Foxp3, Forkhead box Protein 3; MDSC, Myeloid-derived suppressor cell; NOS2, Nitric Oxide Synthase 2; TNFα, Tumor necrosis factor-alpha; IL-2, Interleukin 2; IL-6, Interleukin 6; iNOS, Inducible nitric oxide synthase; M1, Macrophage 1; M2, Macrophage 2; PD-1, Programmed cell death protein 1; PD-L1, Programmed cell death protein ligand 1; CTLA-4, Cytotoxic T-lymphocyte antigen 4; NK, Natureal kill cell; TGF-β, Transforming growth factor-beta; MMP-9, Matrix metallo proteinase-9; VEGF, Vascular endothelial growth factor; **┤**,Inhibition; →, Promotion.

The immune system is vital for the human to defect various pathogens causing infections or tumors with the cooperation of immune cells and some cytokines. As tumor immunotherapy has achieved great success in clinical, the importance of cancer immunotherapy has been gradually acknowledged in these decades. In 2018, the Nobel prize for physiology or medicine was awarded to the Nobel Laureates who found two immune checkpoints: cytotoxic T-lymphocyte associated protein (CTLA-4) and programmed cell death protein 1 (PD-1) and its ligand (PD-L1) (53) that are responsible for the tumor immune evasion. Currently, some tumor immunotherapy agents applied in the treatment of melanoma, lung cancer, head and neck squamous cell cancer have been approved by Food and Drug Administration (FDA) and European Medicines Agency (EMA) (54). However, due to the complicacy of the immune response in the tumor microenvironment (**Figure 3**), further and more studies still should be carried out.

Immunotherapy is a practical strategy to treat osteosarcoma. Immunotherapy enables an increase in the survival rate of patients suffering from osteosarcoma. Back in 1891, Coley’s research manifested that around 10% of patients with bone and soft tissue sarcomas got benefit from the stimulated immune system by the injection of two kinds of heat-inactivated bacteria (55); in a randomized phase III study, Mifamurtide with chemotherapy performed better than monotherapy. In this study, Mifamurtide was used to activate some innate immune cells (e.g. monocytes and macrophages) to control the tumor development as it is an analog of bacteria cell walls (56). Furthermore, adoptive T cell therapy in osteosarcoma also worked well (57, 58). Tumor-infiltrating lymphocytes (TIL) are detected in osteosarcoma by an immunohistochemical study, and among those TILs, CD8+ T-lymphocytes dwarf others (59, 60). Similarly, Tsukahara and his colleagues also found CD8+ T-lymphocytes play a pivotal role in the suppression response to osteosarcoma (61).

Curcumin can improve tumor immunotherapy targeting PD-1/PD-L1 and CTLA-4. Traditionally, researchers focus on the anti-cancer effect of curcumin on various signal pathways in cancer cells, however, an increasing body of literature has indicated that curcumin can elevate no matter the innate or adaptive immune response to cancer (62) by the modulation of T cells, macrophages, dendritic cells (DC), natural killer cells (NK), cytokines, etc. (63, 64). Curcumin can promote T cells quantitively and functionally (62, 65–67). The potential mechanism may include the downregulation of Treg and the expression of some immune checkpoints (e.g. PD-1/PD-L1, CTLA-4). It is well-known that Foxp-3^+^ Treg can suppress cytotoxic T lymphocytes (CTLs) (68), while curcumin has been found to inhibit the activity of Treg *via* the decrease of IL-2 (69). On the other hand, the overexpression of PD-1/PD-L1 and CTLA-4 is responsible for the exhaustion of CTLs, which leads to tumor immune evasion finally. In some previous research, CTLA-4 and PD-1/PD-L1 are identified to overexpress in osteosarcoma and negatively correlate to the prognosis (70–75). Blockade of PD-1 or CTLA-4 can contribute to the inhibition of osteosarcoma, but, in a phase II trial, only 5% of patients with osteosarcoma were relieved by PD-1 inhibitor-pembrolizumab (76). Reassuringly, Taeko et al. found curcumin can enhance the PD-1 blockade therapy (77). Similarly, Paul also found curcumin can improve anti-PD1 efficacy *in vivo* (78). This means combining curcumin with immune checkpoints blockade is a potential promising clinical approach in the treatment of osteosarcoma.

Except for T lymphocytes, some innate immune cells are also of great importance for immunotherapy. Dendritic cell (DC) is a professional antigen presentence cell (APC). With this property, it can activate lymphocytes, not only T cells but also NK, thereby fighting against tumor cells (79). The application of DCs to inhibit some pediatric solid tumors including osteosarcoma has been reported in a clinical study (80). Kawano et al. found DCs pulsed with tumor lysate cannot enhance IFN-γ level in serum and the accumulation of CTLs in metastatic areas (81). What’s more, they also found combining with CTLA-4 blockade in a mouse osteosarcoma model, the anticancer effect had been enhanced: more CTLs, less Treg, prolonged survival, etc. (82). Another immune checkpoint PD-L1 also expresses on DCs and can attenuate T cell activation (83). It has been identified that PD-1 inhibitor combing with DCs vaccines has improved anticancer effect (84, 85). As mentioned above, curcumin may affect the expression of PD-1/PD-L1, according to this, curcumin combing with DCs may also be a promising therapeutic strategy. Interestingly, PD-1 inhibitors also can induce macrophage polarization from M2 (pro-tumor) to M1 (anti-tumor) in an osteosarcoma model (73). In line with this, curcumin may also be able to inhibit osteosarcoma *via* the polarization of macrophages from M2 to M1. Nevertheless, these trials have not been conducted widely, currently.

Taken together, curcumin may modulate the immune response to osteosarcoma by affecting various immune cells, cytokines, and molecular markers, which confers it to be a promising agent for immunotherapy in osteosarcoma.

### Combination With Chemotherapy

Chemotherapy plays a great role in the treatments of tumors, particularly for extensive metastatic advanced ones that cannot be removed by surgical resection. To date, various chemotherapy regimens have been administrated clinically (e.g.. cisplatin, doxorubicin, 5-fluorouracil, methotrexate), and among them, cisplatin is the most used (86). These drugs perform anti-cancer activities through various mechanisms: damaging DNA, activating TP53, increasing the intracellular reactive oxygen species (ROS) level, etc. However, these chemotherapeutic agents are like a “double-edged” sword; they damage both cancer cells and normal somatic cells in the same way, terming as on-target toxicity (87). According to a great amount of previous research, cisplatin and doxorubicin have been confirmed to be toxic to many organs, especially the kidney and heart (88, 89), respectively. Another challenge for the current chemotherapy is multidrug resistance (MDR) impedes the efficacy of chemotherapeutic drugs regarding the activation of NF-κB, overproduced P-glycoprotein (P-gp), etc. (90–92). To overcome this issue, the strategy of escalating dose and group combination has been presented. Nevertheless, this means more toxicity to patients.

ROS plays a crucial role in on-target toxicity and MDR. For on-target toxicity, most chemotherapeutic agents will upregulate the intracellular ROS. Afterward, the accumulated ROS will damage DNA and proteins, and cell membranes, thereby inducing cell apoptosis. Normal cells will also be killed due to oxidative stress in this process. Cisplatin-induced kidney injury and doxorubicin-induced cardiotoxicity are reported to be relative to ROS (93, 94). On the other hand, upgraded ROS can active NF-κB following activation of some chemoresistance genes such as hypoxia-inducible factor 1 alpha and P-gp (95).

Regarding the role of ROS in chemotherapy, combining with antioxidants seems an appealing approach to protect normal cells and circumvent the chemoresistance simultaneously (96–98). Curcumin reverses chemotherapy resistance, which has also been reported. Ehherth et al. found curcumin sensitized CE/ADR5000 cell line from 883-fold doxorubicin-resistance to 0.9-fold (99). As mentioned above, curcumin is a safe natural antioxidant (maximum 12 g/day over 3 months) (100), with the application of it in chemotherapy, there may be an improved synergistic effect and can protect the normal tissues; it is implied that curcumin protects against doxorubicin toxicity (101); the protective effects can also be found in combination with cisplatin (102). Except for ROS, Ma reported that curcumin can increase the absorption of doxorubicin *in vivo* by inhibition of drug efflux, thereby enhancing the chemotherapy efficacy (103). This means curcumin may play a versatile role in the combination with chemotherapeutic agents.

With the introduction of chemotherapy in osteosarcoma treatment, long-term survival rates have increased from less than 20 to 65–70%, and the first-line drugs are MAP: methotrexate, doxorubicin, and cisplatin (104). However, the survival of patients bearing osteosarcoma has not been improved since the last decades, although chemotherapy strategy for osteosarcoma has developed: neoadjuvant and adjuvant chemotherapy. To enhance the chemotherapy efficacy, numerous studies with the addition of some drugs to MAP have been conducted, however, data from these studies did not show any improvement. A trial started in 2005 conducted by the European and American Osteosarcoma Study Group showed the addition of interferon-alpha in neoadjuvant chemotherapy, and ifosfamide and etoposide in adjuvant chemotherapy did not show a statistical difference (105); and the French multicenter OS2006 added zoledronic to chemotherapy, there was no significant enhancement either (106). Other agents (topotecan, imatinib, oxaliplatin, ixabepilone, etc.) tested in selected phase II trials in osteosarcoma did not show any positive results (104). Although curcumin seems a drug with great synergistic effort in chemotherapy, there is little research about this strategy in the chemotherapy of osteosarcoma until now. Further investigations about this strategy are required in the future.

### Combination With Bone Tissue Engineering Materials

To remove the primary tumor thoroughly, an extended resection area is the main approach currently. In osteosarcoma treatment, this may cause critical size bone defects, while insufficient resections are always responsible for the tumor recurrence. This seems to be in a dilemma. To repair the critical size bone defects (more than 2 cm, typically), autografts and allografts are prevailing strategies, and autografts are considered to be the “gold standard” (107). Nevertheless, the application of autografts and allografts will cause some side effects. Autografts may cause the morbidity of donor sites (pain, hematomas, nerve injuries, etc.); allografts may result in disease transmission. To overcome these issues, various biomaterials have been developed and applied clinically, among which the prevailing materials are polymers (natural or synthetic), bioceramic, and composite materials (108). These materials achieve great success in osteogeneration. However, most of these materials lack the anti-cancer property, which means they are ineffective for potential tumor recurrence. The combination of curcumin with these materials is a promising strategy to resolve this problem. As mentioned above, curcumin cannot suppress osteosarcoma development but induce osteogenesis. The addition of curcumin can promote bone repairment and protect against the potential remaining carcinoma. Naboneeta documented that curcumin loaded with hydroxyapatite-coated Titanium implant enhanced the cytotoxicity to MG-63 *in vitro* (109). In another study, he and his colleagues pointed out that curcumin loaded on 3D printed calcium phosphate scaffold presented selective toxicity to MG-63 cells and promoted normal osteoblast proliferation (110). Another benefit of this combination strategy is increasing the accumulation of curcumin in lesions. Due to extensive first-pass metabolism and poor curcumin bioavailability (111), traditional delivery methods are powerless to overcome these issues. Loading in/on these materials, curcumin can accumulate in the target area directly, therefore, its pharmacological efficacy boost.

To refine the stability and bioavailability of curcumin, some nanoparticles are used. In a review, encapsulating curcumin in liposomal nanoparticles, the most used way, improved its anticancer efficacy (112). Currently, some more sophisticated combination strategies have been proposed. The chemotherapeutic drug, photosensitizer, and immune checkpoint blockade were loaded in the same nanoscale polymers, by which the anticancer effect increased significantly (113). Based on this, curcumin, a versatile agent with all these properties, is a prospective candidate in a nano delivery system.

## Prospect of Application of Curcumin in Photodynamic Therapy

Photodynamic therapy (PDT) is an emerging treatment modality. To date, it has been applied in many fields including dermatology, oncology, gynecology, and urology (114). It is thought to be a non-invasive remedy, as it kills pathogens or tumor cells depending on the phototoxicity resulting from the intracellular accumulation of ROS attributing to the “photodynamic effect” referred to in 1904 by Von Tappeiner (115). The production of exceeded ROS is based on the mutual interaction among the photosensitizers (PS), light with appropriated wavelength, and intracellular oxygen molecules. There are two types of reactions in PDT with the same initiation- exciting PS using appropriated light. Afterward, the excited PS may transport electrons to cellular substrates (Type I reaction) or molecular oxygen directly (Type II reaction) (116). In the former, free radicals and anion radicals (hydroxyl radical, and superoxide ion) were generated, and singlet oxygen was found in the latter, which is considered to be the most dangerous one among ROS as it can react with unsaturated lipids, proteins (117) with its potent oxidative property, thereby damaging the cell and nuclear membranes (118).

**Figure 4:**
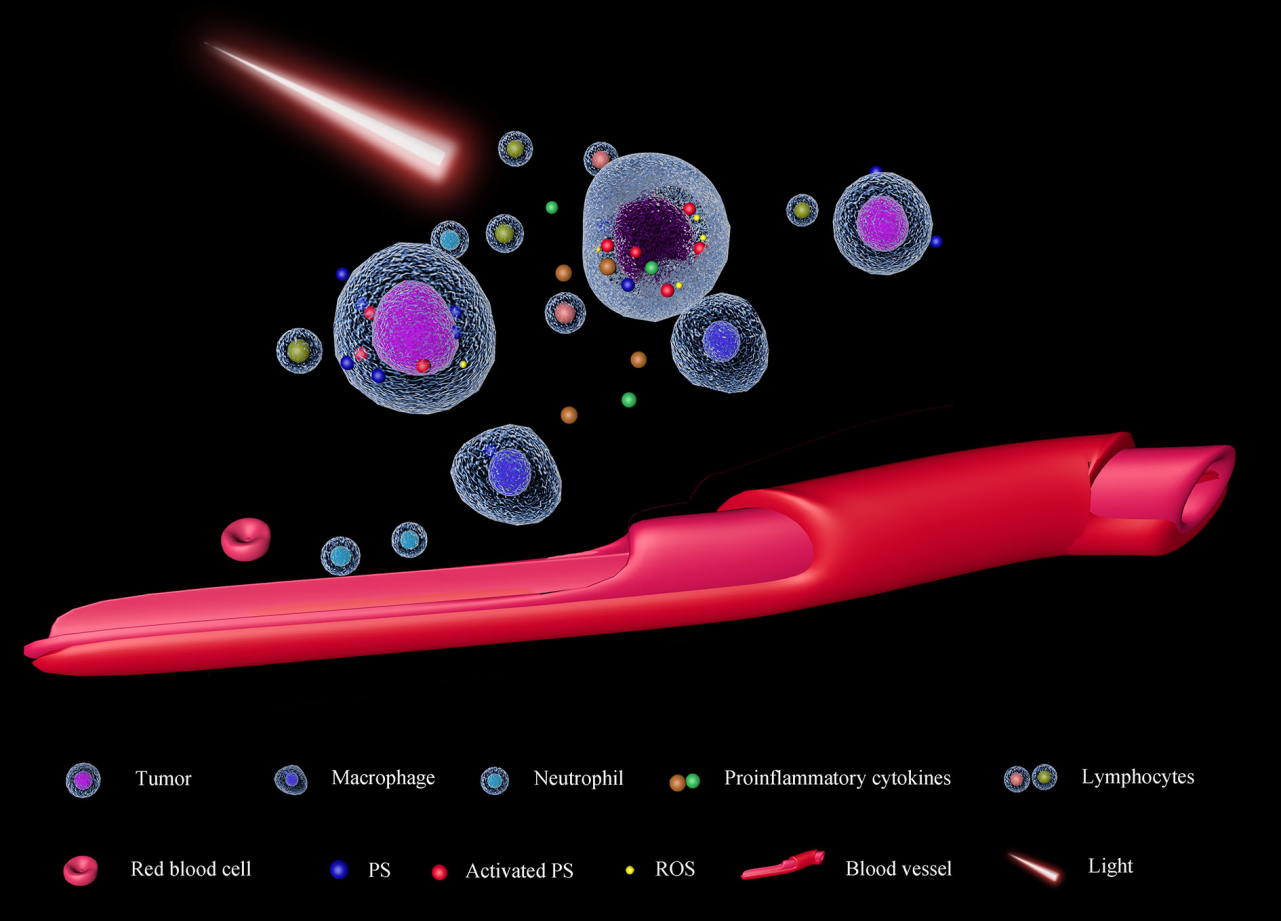
Schematic diagram of PDT. The photosensitizer (PS) will be activated by light with an appropriate wavelength, causing the accumulation of ROS in the cells. Exceeded ROS damages tumor cells directly and epithelial cells of tumor-associated vessels. As the impairment of the tumor cell membrane, some pro-inflammatory cytokines will be released, facilitating the recruitment of immune cells in the tumor microenvironment (TME). On the other hand, as the tumor-associated vessels are also damaged, nutrients and oxygen supplements for the tumor will decrease; moreover, the neutrophils and macrophages can transport into the TME, suppressing tumor proliferation.

PDT was firstly approved in Canada in 1993 for the therapy of bladder cancer (119), and more than 200 clinical trials have been carried out. Photofrin, a first-generation and most used PS has been approved to treat cancers by FDA (120) and it is still used now. The anticancer effect of PDT is based on these mechanisms: direct killing cancers by ROS, inhibiting the angiogenesis (121), and activating the immune system (122) (**Figure 4**). ROS can cause the death of cancer cells and vascular endothelial cells. In this condition, oxygen and nutrition supplements for tumors will be dampened, causing cancer cell death. Afterward, some pro-inflammatory cytokines will be released to recruit and activate immune cells (123). The broken vascular walls also facilitate the recruitment of neutrophils and macrophages in the tumor microenvironment (124). Additionally, Castano et al. also found PDT can suppress the Treg (125) which always silences cytotoxicity T lymphocytes. Based on these, a combination of PDT with immune checkpoint inhibitors may enhance the anti-cancer effect. In a case report, a patient with advanced head and neck squamous cell cancer received radiotherapy, surgery, and chemotherapy, which did not control the development of cancer. Afterward, with PDT, the visible tumor vanished, and combining PD-1 blocker, the patient was with no signs of the disease two years later (126).

Although curcumin, to date, has not been applied in the treatment of osteosarcoma clinically, curcumin has been found that it can work as a PS in PDT with enhanced anti-cancer or anti-bacteria effect (127, 128). Curcumin although is a phytochemical agent, its chemical structure is declared clearly. It is available to obtain highly pure commercial production and meet the potential tremendous clinical need. Besides, regarding the non-toxicity of curcumin to normal tissues (100), curcumin used in PDT can reduce the potential damage to normal cells. Moreover, to reduce the damage to normal tissues, the selectivity of PS is also crucial. Ideally, the more PS distributed in tumors, the better efficacy, and fewer side effects can be induced. It has been proved that tumor takes up more curcumin than normal cells (129). All these suggest curcumin is a promising PS, while it also has a great challenge in clinical application. To excited PS, light with an appropriated wavelength is vital. The optimal wavelengths are between 600 and 850 nm, termed as “therapeutic windows”, as the lower ones cannot penetrate deep tissues and higher ones without sufficient energy cannot excite PS to generate singlet oxygen (130). Unluckily, the Ex of curcumin is just around 425 nm (131), which is cannot penetrate skins to excite curcumin in osteosarcoma PDT. To overcome this problem, using a fiber optic device may be a practical approach. Another drawback of curcumin-hydrophobicity also dampens its efficacy in PDT. It is documented that PS can perform photoactive only in the monomeric form (132). Curcumin will aggregate in an aqueous environment, reducing its excitation. These disadvantages may be contributed to the limitation of its clinical trials. More advantages and modifications of curcumin are in high demand to adjust to the PDT.

## Conclusion

Curcumin, a multifunctional phytochemical, has been identified to be a promising anticancer drug based on abundant *in vitro* and *in vivo* studies. Nonetheless, due to its hydrophobicity, poor bioavailability, there are few clinical trials demonstrating comforting results, neither successful clinical applications. For osteosarcoma treatment, most of the current research about the effect of curcumin is carried out *in vitro*, which may weaken the comforting results from these studies. Established OS animal models using different OS cell lines have been reported, while few of them have been applied to test curcumin resulting from its inherent disadvantages that may affect the feasibility and impede the accurate assassination. To circumvent this limitation and provide more reliable conclusions from no matter cellular and animal research or pre/clinical trials, more measures have to be implemented. On one hand, chemical modification of curcumin or analogs has been carried out to enhance its solubility in water and bioavailability in physiological conditions. On the other hand, the combination of curcumin with other therapeutic strategies is also promising. Thanks to its versatile properties, curcumin can improve chemotherapy and immunotherapy efficiency. Moreover, curcumin can also work as a photosensitizer in PDT. Interestingly, these three approaches can work synergistically. In line with this, curcumin may combine a wide range of agents as a sophisticated systemic strategy to suppress oncogenesis. In osteosarcoma remedy, curcumin loaded in bone-engineering materials can inhibit osteosarcoma cells and promote osteogenesis simultaneously. This property makes curcumin stand out from a great variety of anticancer drugs. In this approach, bone-engineering materials not induce osteogenesis but work as a controlled delivery system of curcumin that enhances the local concentration of curcumin and prolongs its duration of action. Taken together, although curcumin has a great anticancer property, to widen its clinical application, more modifications and further studies are still required.

## Author Contributions

CX contributed to the conception of this work and drafted the manuscript. MW collected literature. WG and WS made important revisions and polished the language. YL edited and revised this manuscript and approved the publication. All authors contributed to the article and approved the submitted version.

## Conflict of Interest

The authors declare that the research was conducted in the absence of any commercial or financial relationships that could be construed as a potential conflict of interest.
